# A Novel Defined Super-Enhancer Associated Gene Signature to Predict Prognosis in Patients With Diffuse Large B-Cell Lymphoma

**DOI:** 10.3389/fgene.2022.827840

**Published:** 2022-06-14

**Authors:** Hong Xu, Yuhang Li, Yanan Jiang, Jinhuan Wang, Huimeng Sun, Wenqi Wu, Yangyang LV, Su Liu, Yixin Zhai, LinYan Tian, Lanfang Li, Zhigang Zhao

**Affiliations:** ^1^ Department of Hematology, Key Laboratory of Cancer Prevention and Therapy, National Clinical Research Center for Cancer, Tianjin’s Clinical Research Center for Cancer, Tianjin Medical University Cancer Institute and Hospital, Tianjin, China; ^2^ Department of Oncology, Institute of Urology, Second Hospital of Tianjin Medical University, Tianjin, China; ^3^ Departments of Lymphoma, Tianjin Medical University Cancer Institute and Hospital, National Clinical Research Center of Cancer, Key Laboratory of Cancer Prevention and Therapy, Tianjin's Clinical Research Center for Cancer, Tianjin, China

**Keywords:** super-enhancer, LASSO, diffuse large B-cell lymphoma, prognostic model, overall survival

## Abstract

**Background:** Diffuse large B-cell lymphoma (DLBCL) is a genetically heterogeneous disease that can have profound differences in survival outcomes. A variety of powerful prognostic factors and models have been constructed; however, the development of more accurate prognosis prediction and targeted treatment for DLBCL still faces challenges. An explosion of research on super-enhancer (SE)–associated genes provide the possibility to use in prognostication for cancer patients. Here, we aimed to establish a novel effective prognostic model using SE-associated genes from DLBCL.

**Methods:** A total of 1,105 DLBCL patients from the Gene Expression Omnibus database were included in this study and were divided into a training set and a validation set. A total of 11 SE-associated genes (BCL2, SPAG16, PXK, BTG1, LRRC37A2, EXT1, TGFBR2, ANKRD12, MYCBP2, PAX5, and MYC) were initially screened and identified by the least absolute shrinkage and selection operator (Lasso) penalized Cox regression, univariate and multivariate Cox regression analysis. Finally, a risk score model based on these 11 genes was constructed.

**Results:** Kaplan–Meier (K–M) curves showed that the low-risk group appeared to have better clinical survival outcomes. The excellent performance of the model was determined *via* time-dependent receiver operating characteristic (ROC) curves. A nomogram based on the polygenic risk score was further established to promote reliable prognostic prediction. This study proposed that the SE-associated-gene risk signature can effectively predict the response to chemotherapy in DLBCL patients.

**Conclusion:** A novel and reliable SE-associated-gene signature that can effectively classify DLBCL patients into high-risk and low-risk groups in terms of overall survival was developed, which may assist clinicians in the treatment of DLBCL.

## Introduction

Diffuse large B-cell lymphoma (DLBCL) is the most common type of non-Hodgkin’s lymphoma (NHL), accounting for 30%–40% of all newly diagnosed NHL cases (Armitage et al., 2017; Siegel et al., 2017). DLBCL is an aggressive, severe, and complex disease with broad genetic, phenotypic, and clinical heterogeneities (Abramson and Shipp, 2005). The heterogeneity of the disease results in different survival outcomes in DLBCL patients receiving standard therapy (rituximab, cyclophosphamide, doxorubicin, vincristine, and prednisone (R-CHOP)) (Younes, 2015). About 30–40% of patients do not respond well to standard treatment, with the highest mortality rate in the first 2 years after diagnosis(Yin et al., 2019).

In the era of rituximab, the International Prognostic Index (IPI) is one of the most important tools for prognostic risk stratification. The subsequent revisions have appeared to improve the prognostic evaluation system in DLBCL patients. Disappointingly, these prognostic indicators do not address the underlying biological heterogeneity of DLBCL. Therefore, it is urgent to explore novel and effective molecular markers for a more accurate prediction of the prognosis of patients with DLBCL.

Super-enhancers (SEs) have been described as a class of regulatory domains with unusually strong transcription-assisted activator binding capacity (Parker et al., 2013; Whyte et al., 2013). SE is a cluster of enhancers that has a stronger ability to promote transcription compared to the typical enhancers (TEs). Compared with normal cells, tumor cells construct SEs on oncogenes during tumorigenesis and recruit enhancer-binding proteins to drive gene expression (Lovén et al., 2013). SEs are generally occupied with abundant signals of H3K4me1, H3K27ac, p300, Mediator, RNA polymerase II, BRD4, CDK7, and other master transcription factors (Wang et al., 2019); among them, H3K27ac is the preferred marker for the identification of super-enhancers (Hnisz et al., 2013). The loss or gain of SEs has been reported in various tumors (He et al., 2019); similarly, SEs play a key role in the progression of DLBCL by activating the expression of downstream oncogenes (Chapuy et al., 2013). In addition, SE inhibitors (JQ1) used to treat DLBCL suppress the expression of these genes (Li et al., 2021). Therefore, the exploitation and identification of SEs-driven hub oncogenes will provide novel insights into the diagnosis, prognosis, and treatment of DLBCL.

The least absolute shrinkage and selection operator (Lasso) penalized Cox regression is a variable selection and contraction method in Cox’s proportional risk model proposed by Tibshirani (1997). Lasso can reduce the number of variables compared to traditional stepwise regression because less influential variables will be regularized by shrinking their coefficients to zero (Zhang et al., 2018). Currently, Lasso is widely used to build survival prediction models based on complex, high-throughput genomic data. Wu et al. (2021) identified ten important immune-related genes most associated with the overall survival of DLBCL patients among the 26 immune-related genes by using Lasso regression analysis. Similarly, using group Lasso, an 11-SE-related-gene signature effectively predicted overall survival in DLBCL. Thus, we applied the Lasso regression method to construct a prognostic model of DLBCL.

**FIGURE 1:**
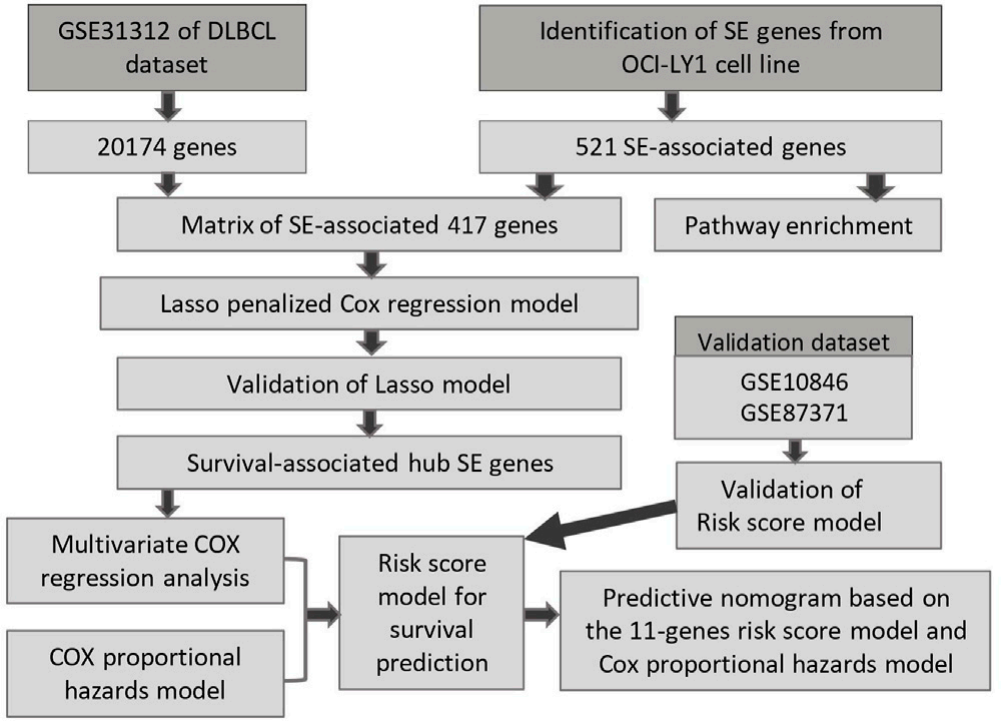
The procedure workflow used to establish and certify the SE-associated gene-based prognostic model for patients with diffuse large B-cell lymphoma.

In this study, Lasso penalized Cox regression analysis was performed using 521 SE-associated genes. A gene cluster containing 11 SE-related genes (BCL2, SPAG16, PXK, BTG1, LRRC37A2, EXT1, TGFBR2, ANKRD12, MYCBP2, PAX5, and MYC) was screened. Subsequently, a risk score model based on these 11 genes was constructed, which was helpful for risk stratification and prognosis. Finally, based on the model, an interactive nomogram containing 11 gene risk groups and clinical characteristics was established, which provides a tool to predict the overall survival (OS) of DLBCL patients clinically. The workflow of our study is shown in Figure 1.

## Materials and Methods

### Data Source

The microarray data and corresponding clinical information from GSE31312 as the training data and the two other independent datasets, GSE10846 and GSE80371, as the external validation datasets were obtained from Gene Expression Omnibus (GEO) database. 470 DLBCL samples were enrolled in GSE31312, 414 in GSE10856, and 221 in GSE80371.

### Identification of Super-Enhancer–Associated Genes

The 521 SE-associated genes identified from the DLBCL cell line OCY-LY1 were obtained from the website http://dbcorc.cam-su.org. H3K27ac chromatin immunoprecipitation sequencing (ChIP-seq) signal was used to screen SE-associated genes in the OCY-LY1 cell line. The biological function of these genes was revealed by Gene Ontology (GO) enrichment and Kyoto Encyclopedia of Genes and Genomes (KEGG) pathway enrichment analysis. To get the final expression matrix, we retained the genes that overlapped between GSE31312 datasets and the SE-associated genes in OCY-LY1.

### Lasso Penalized Cox Regression Analysis

To screen the important and potential prognostic genes, Lasso penalized Cox regression analysis was performed to establish a predicting model using the R package “glmnet”. We identified the optimal lambda (λ) value based on ten-fold cross-validation. Two best-fit values (λ_min_ and λ_lse_) were chosen by minimizing the mean cross-validated error to construct the Lasso models. Subsequently, we performed the Wilcoxon test and ROC curve analysis to compare the two parameters.

### Development of the Prognostic Signature

To construct an optimal prognostic prediction model, we integrated the candidate genes’ expression levels weighted by their regression coefficients and calculated the risk score for each patient, according to the forum RiskScore = ∑βi * Xi. Here, Xi is the gene expression level, and βi is the regression coefficient. Regarding the value obtained from the maximally standardized long-rank statistics as a cutoff point, DLBCL patients were separated into high- and low-risk groups.

### Cox Proportional Hazard Regression Analyses

The univariate and multivariate Cox proportional hazard regression models were utilized to identify the correlation between the gene expression level of the candidate genes and OS, which was accomplished by R packages “survival” and “survimer”. The results were shown on the forest plot. The analyses were also applied to verify the independence of the constructed prognostic model with other clinical features. The parameters included the prognostic risk score and some important clinicopathological factors, such as age, gender, clinical stage, the situation of extranodal invasion, Eastern Cooperative Oncology Group (ECOG) score, lactate dehydrogenase (LDH), and IPI score. The *p-*value, hazard ratio (HR), and 95% confidence interval (CI) of each factor were calculated.

### Kaplan–Meier Analysis and Time-dependent Receiver Operator Characteristic Curve Analysis

The Kaplan–Meier analysis method was used to compare the differences in OS and progression-free survival (PFS) between low- and high-risk groups, and the log-rank tests were performed to measure the statistical significance (*p*-value of less than 0.05). The R packages “survival” and “survimer” were used to execute the analysis. Moreover, we depicted the time-dependent ROC curve to assess the predictive capability for different factors by figuring out the area under the ROC (AUC) (*p* < 0.05).

### Predictive Nomogram

In total, seven prognostic predictors (six clinical features and the 11-genes risk score) were enrolled to build the predictive nomogram, which was used to forecast the 1-year, 3-year, and 5-year OS of the patients *via* R package “rms”. We calculated the concordance index (C-index) by package “Hmisc” to evaluate the discrimination of the nomogram. Furthermore, calibration curves were plotted for intuitionistic comparison of the predicted against the actual survival probabilities. Data of one randomly selected patient from GSE31312 were used to validate the probability of 1–5-year OS, based on the predictors in the nomogram. Total points were calculated using the R package “nomogramEx”. Finally, the interactive nomogram was developed and visually displayed by the R package “regplot”.

### Chemotherapy Response With Super-Enhancer-Associated Genes Signature

In order to predict the chemotherapy response in the low- and high-risk groups, the R package “pRRophetic” was applied for profiling. We straightforwardly compared the estimated half-maximal inhibitory concentration (IC_50_) between low- and high-risk groups among the different chemotherapeutics, which exactly proved the hypothesis that the low-risk group was likely more sensitive to the chemotherapy.

### Protein–Chemical Interactions Analysis and Chromatin Immunoprecipitation Sequencing Profile for H3K27ac Signal Tracks

We established an interactive network of the hub genes and chemicals to probe into the chemicals correlated to these genes by “NetworkAnalyst 3.0”, based on the data from the Comparative Toxicogenomics Database (CTD). In the end, we used H3K27ac as SE biomarkers based on the ChIP–seq profiles data from Cistrome to visualize the location of the SEs regions and their target genes.

## Result

### Establishment of the Lasso Penalized Cox Regression Model

**FIGURE 2:**
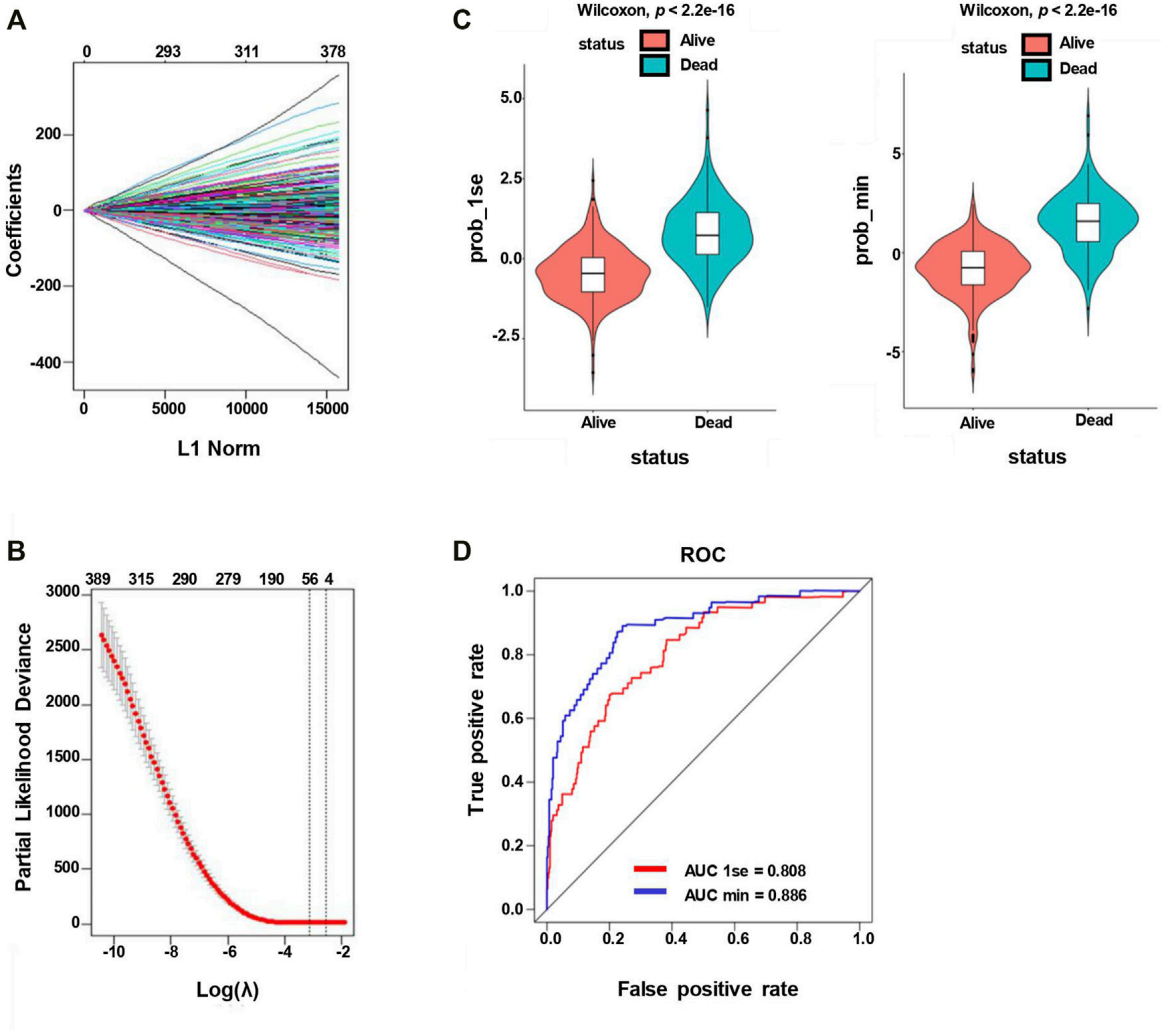
Lasso penalized Cox regression analysis of SE-associated 512 genes. **(A)** Lasso coefficient profiles of the 512 SE-associated genes. **(B)** The identification of the best Lambda value. The left solid vertical line is the logarithm of lambda.min (48-gene group), and the right solid vertical line is the logarithm of lambda.1se (16-gene group). **(C)** The scatter plot of survival status of patients with diffuse large B-Cell lymphoma based on the 48-gene model (left, lambda.min, *p* < 2.2e−16) or the 16-gene model (right, lambda.1se, *p* < 2.2e−16) by the Wilcoxon test. **(D)** ROC curves are used to compare the predictive performance for prob-min and prob-1se to predict patient survival.

A 20,174-gene expression matrix of GSE31312 and the corresponding clinical information of 470 DLBCL patients were downloaded from the GEO database under the accession number GSE31312, as described in Supplementary Table S1. In total, 521 SE-associated genes identified from the DLBCL cell line OCY-LY1 were obtained from the website http://dbcorc.cam-su.org. Pathway enrichment analysis indicated that these SE-associated genes were closely related to lymphocyte activation and small GTPase mediated signal transduction (Supplementary Figures S2A and C). We extracted 417 genes that overlapped between GSE31312 datasets and the SE-associated genes in OCY-LY1 to construct the expression matrix. The lasso penalized Cox regression analysis was applied to screen some potential and vital prognostic genes. We calculated the coefficient values at different levels of penalty (Figure 2A). First, we identified the optimal lambda (λ) value based on ten-fold cross-validation. Two best-fit values (lambda.min and lambda.1se) were chosen by minimizing the mean-square error to construct the Lasso models, and we selected two groups of genes (48-gene group of λ_min_ and 16-gene group of λ_1se_; Figure 2B). As shown in Figure 2C, the lasso models were reconstructed according to the λ_min_ and λ_lse_, and both models performed well to separate the survival and death events (Wilcoxon test, *p <* 2.2e-16). The result of the ROC curves analysis for the two predictive models showed the AUCs were 0.808 (λ_1se_) and 0.886 (λ_min_), suggesting that both models had a promising performance in predicting the probability of overall survival (Figure 2D). Considering that there was no significant difference in the predictive performance of the two models according to AUC and Wilcoxon tests, we further studied the 16-gene model.

### Association Between Candidate Genes and Prognosis

**FIGURE 3:**
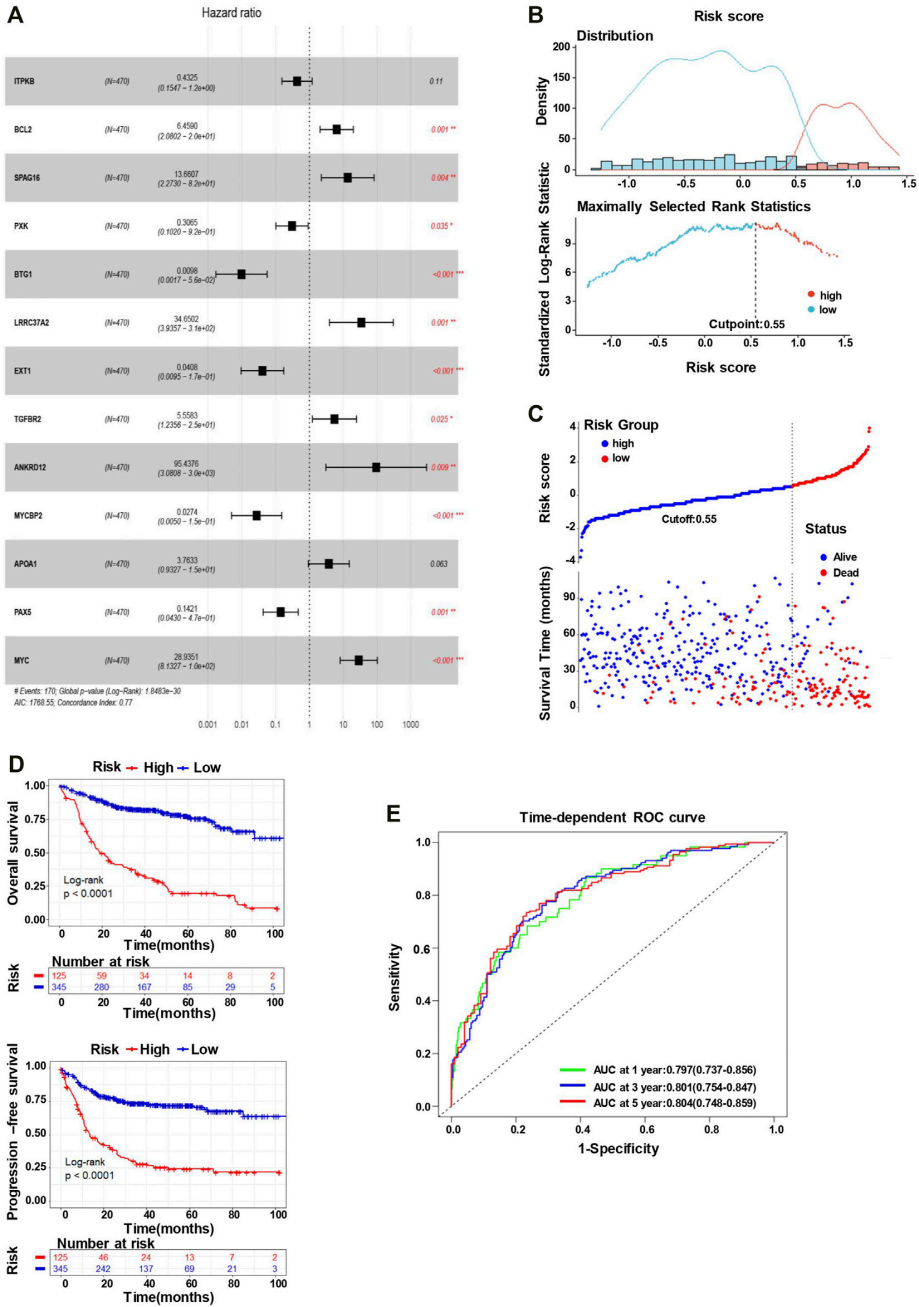
The 11-gene risk score model for the GSE31312 dataset. **(A)** Multivariate Cox regression analysis of the 13 genes (*p < 0.05, **p < 0.01, and ***p < 0.001). Hazard ratio and 95% CI are shown in the figure. Global log-rank *p*, C-index, and AIC were also calculated and shown. **(B)** The identification of the cutoff value (cutpoint=0.55) of the risk score. **(C)** DLBCL patients were divided into the high-risk group and low-risk group based on the cutoff value (upper). The survival status and time in high-risk and low-risk groups (lower). **(D)** Kaplan–Meier survival curves showing the difference in OS (upper) and PFS (lower) between high- and low-risk patients (log-rank test, *p* < 0.0001). **(E)** Time-dependent ROC curves for the 11-gene model to predict patient survival.

We utilized multivariate Cox regression analyses to explore whether each of the candidate genes is associated with the overall survival. As the outcome of the multivariate Cox regression analysis shown in Figure 3A, the global *p*-value of the predictive model was 1.8483e-30, with the Akaike information criterion (AIC) of 1768.55 and C-index of 0.77. Multivariate Cox regression showed that BCL2, SPAG16, PXK, BTG1, LRRC37A2, EXT1, TGFBR2, ANKRD12, MYCBP2, PAX5, and MYC were significantly associated with the overall survival of DLBCL patients. Among these genes, BCL2, SPAG16, LRRC37A2, TGFBR2, ANKRD12, and MYC may appear to be the risky factors (HR > 1), while PXK, BTG1, EXT1, MYCBP2, and PAX5 seemed to act as the protective factors (HR < 1). To optimize the predictive model, we selected these 11 SE-associated genes to forecast the OS of DLBCL patients.

### Establishment and Validation of the 11-Gene Risk Score Model

The risk scores predicted by the coefficient of these 11 candidate genes from the multivariate Cox regression analysis (the equation for risk scores is shown in Materials and Method) stratify the patients into the low-risk (*n* = 345) and high-risk (*n* = 125) groups, with the cutoff point of 0.55 (Figure 3B). As the outcome shown, the number of alive events is significantly more in the low-risk group, while the death events are obviously more frequent in the high-risk group (Figure 3C). Subsequently, we conducted a K–M analysis to compare the differences in OS and PFS between low- and high-risk groups. The K–M survival curve of OS demonstrated an inferior outcome in the high-risk group (long-rank test, *p* < 0.0001), consistent with the analysis of PFS (Figure 3D). Furthermore, the time-dependent ROC analysis also showed a favorable outcome, where the AUC was 0.797 at 1-year, 0.801 at 3-year, and 0.804 at 5-year (Figure 3E), indicating that the risk score model has a good performance to predict the prognostic outcomes.

### Independence of 11-Gene Risk Score Model in Survival Prediction

**FIGURE 4:**
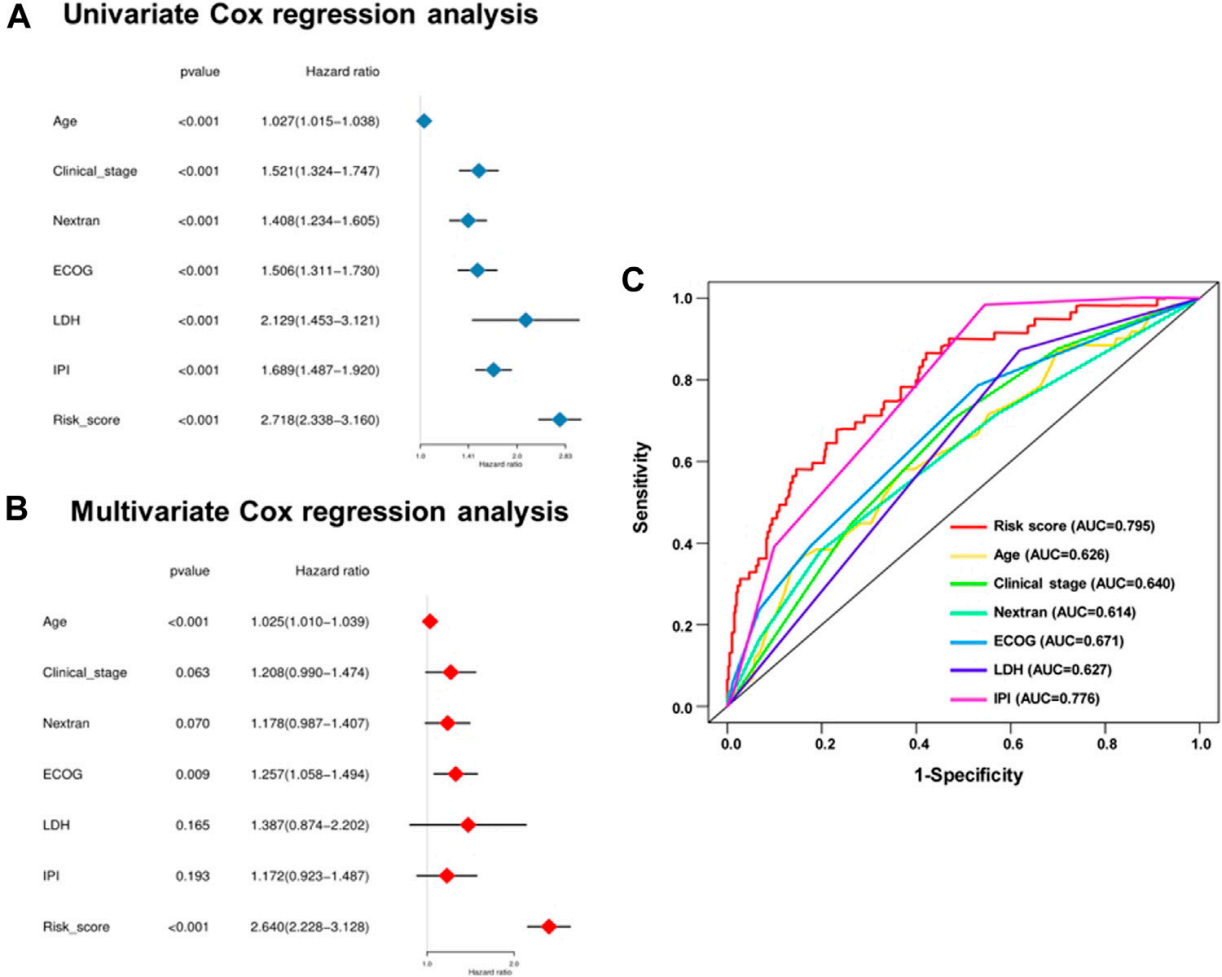
Univariate and multivariate analysis shows the prognostic value of 11-SE-associated-gene signature. Univariate **(A)** and multivariate **(B)** Cox regression analyses of the association between clinicopathological factors and OS of DLBCL patients. **(C)** The receiver operator characteristic (ROC) curves to predict the sensitivity and specificity of clinicopathological factors and 11-SE-associated-gene signature-derived risk scores in DLBCL patients.

Considering the effects of other important clinical indicators, such as age, gender, clinical stage, the situation of extranodal invasion, ECOG score, LDH level, and IPI score, we validated the independence of the polygenic prognostic predictive model *via* the univariate and multivariate Cox regression analyses. In the univariate Cox regression analysis, the risk score correlated with OS of the DLBCL patients (HR at 2.718, *p* < 0.001), similar to other important clinicopathological factors (Figure 4A). As for the multivariate Cox regression analysis, risk score appeared to be an independent and harmful factor for prediction (HR at 2.640, *p* < 0.001), while only Age and ECOG score among all clinical features showed statistical significance (*p* < 0.001 and *p* = 0.009, respectively) (Figure 4A; Table 1). The ROC curve analysis was a complement for verifying the predictive capacity of these indicators, which showed that the AUC of the risk score was 0.795, greater than other clinical indicators (Figure 4B). All these results sufficiently confirmed that our 11-gene risk score model was an independent and robust predictor, which has promising application prospects in comparison with other well-establish indicators.

**TABLE 1:** Univariate and multivariate Cox regression analyses of the gene signature and overall survival of DLBCL patients in 3 independent datasets.

Variables		Patients(N)	Univariate analysis		Multivariate analysis	
			HR (95%CI)	*p* Value	HR (95%CI)	*p* Value
GSE31312						
Age	>60/<=60	270/200	1.027 (1.015–1.038)	<0.001	1.025 (1.010–1.039)	<0.001
Clinical stage	III-IV/I-II	229/220	1.521 (1.324–1.747)	<0.001	1.208 (0.990–1.474)	0.063
Extranodal sites	>=2/<2	104/366	1.408 (1.234–1.605)	<0.001	1.178 (0.987–1.407)	0.07
ECOG	>=2/<2	96/374	1.506 (1.311–1.730)	<0.001	1.257 (1.058–1.494)	0.009
LDH	Evaluated/normal	278/148	2.129 (1.453–3.121)	<0.001	1.387 (0.874–2.202)	0.165
IPI score	>2/<=2	150/274	1.689 (1.487–1.920)	<0.001	1.172 (0.923–1.487)	0.193
Risk score	High/Low	125/345	2.718 (2.338–3.160)	<0.001	2.640 (2.228–3.128)	<0.001
GSE10846						
Age	>60/<=60	226/188	1.030 (1.018–1.041)	<0.001	1.030 (1.016–1.045)	<0.001
Clinical stage	III-IV/I-II	218/188	1.508 (1.293–1.758)	<0.001	1.313 (1.091–1.580)	0.004
Extranodal sites	>=2/<2	30/353	1.206 (1.001–1.452)	0.049	0.956 (0.755–1.210)	0.707
ECOG	>=2/<2	93/296	1.820 (1.551–2.136)	<0.001	1.534 (1.273–1.847)	<0.001
LDH	Evaluated/normal	178/173	1.137 (1.095–1.181)	<0.001	1.116 (1.059–1.177)	<0.001
Risk score	High/Low	126/288	2.718 (2.128–3.472)	<0.001	2.039 (1.542–2.697)	<0.001
GSE87371						
Age	>60/<=60	106/115	1.049 (1.025–1.072)	<0.001	1.010 (0.984–1.036)	0.454
Gender	Male/Female	116/105	1.499 (0.863–2.604)	0.151	1.336 (0.761–2.347)	0.313
Clinical stage	III-IV/I-II	150/71	1.802 (1.297–2.503)	<0.001	0.724 (0.441–1.186)	0.200
IPI score	>2/<=2	102/119	2.029 (1.622–2.538)	<0.001	1.666 (0.921–3.015)	0.092
Risk score	High/Low	79/142	2.718 (1.991–3.711)	<0.001	2.289 (1.690–3.099)	<0.001

### Stratification Analysis

A stratification analysis was carried out to assess the predictive abilities of the risk score model within different clinical feature subgroups. Patients from the entire cohort were factitiously classified by age (>60 vs. <=60), gender (Male vs. Female), disease clinical stage (stage I–II vs. III–IV), the situation of extranodal invasion (extranodal sites >=2 vs. < 2), IPI score (>2 vs. <=2), and disease classification based on immunohistochemical (IHC) [activated B cell (ABC), germinal center B cell (GCB), and unclassified (UC)] as different subgroups. The risk score divided the patients in the same stratum into the low- and high-risk groups. We observed that the K–M curves could be distinguished by the risk score model irrespective of the subgroup, where all the high-risk groups had inferior survival outcomes (Supplementary Figure S1).

### Development of Predictive Nomogram for Prognosis Prediction

**FIGURE 5:**
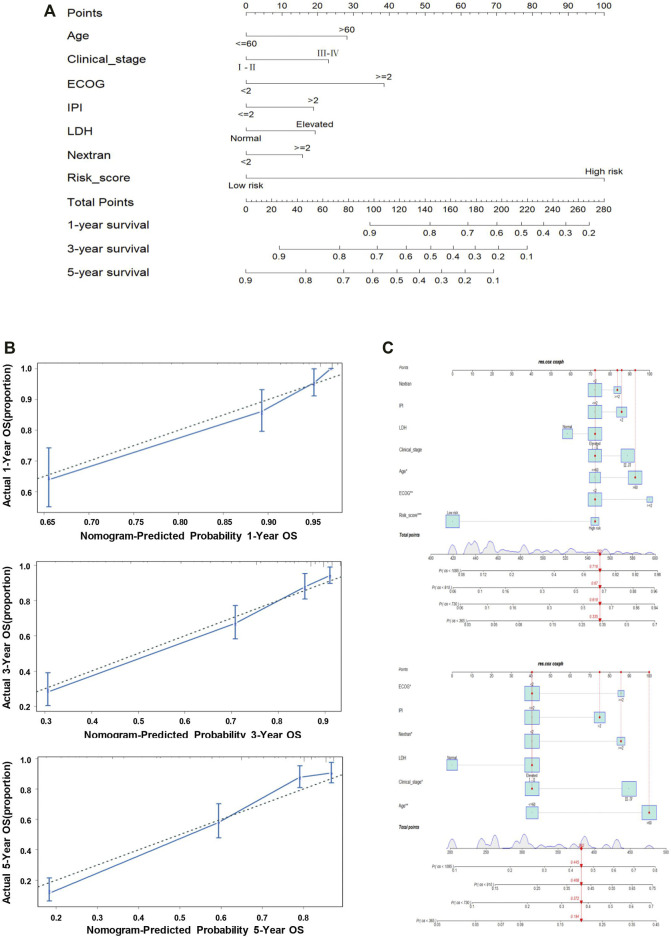
Nomogram predicting the probability of 1-, 3-, and 5-year OS in patients with DLBCL. **(A)** Nomogram adding up the points identified on the points scale (the upward line) for each variable. The total points projected on the bottom scales indicate the probability of 1-, 3-, and 5-year OS. **(B)** Calibration plot for predicting the 1-, 3-, and 5-year OS. The dotted line represents the ideal condition. **(C)** Nomogram predicting the probability of 1-, 3-, and 5-year OS for the specific patient GSM776084 based on the model containing or not containing the risk group in the GSE31312 dataset.

There were seven prognostic predictors enrolled for building the predictive nomogram to forecast the 1-year, 3-year, and 5-year OS for the patients. The predictors of the nomogram involved the 11-genes risk score and the other six clinical indicators: age, clinical stage, ECOG, IPI, LDH, and extranodal sites (Figure 5A). Calibration curves were plotted for intuitionistic comparisons of the predicted against actual survival probabilities. The calibration curves of 1- to 5-year all appeared very close to the grey lines, suggesting a powerful predictive ability of this nomogram (Figure 5B). In order to evaluate the predictive effect of the 11-genes risk score based on the nomogram, we randomly selected one specific patient from the entire cohort. We added up all the points from these clinical indicators and the 11-gene risk group; the total point was 551, compared with the total point of 382 when only considering the clinical variables. The probability of 1-, 3-, and 5-year OS were 0.335, 0.618, and 0.716, respectively, while taking both the clinical indicators and risk group into account. In reality, the patient died at 910 days, while the predictive probability of death at that day was 0.67. Meanwhile, when we only utilized the six clinical indicators, the probability of 1-, 3-, and 5-year OS were 0.194, 0.372, and 0.445, respectively. The predictive probability of death at 910 days was 0.408, obviously lower than the probability forecasted in consideration of the 11-genes risk score, as mentioned above (Figure 5C).

### Validation of the 11-Genes Prognostic Signature in the External Datasets

**FIGURE 6:**
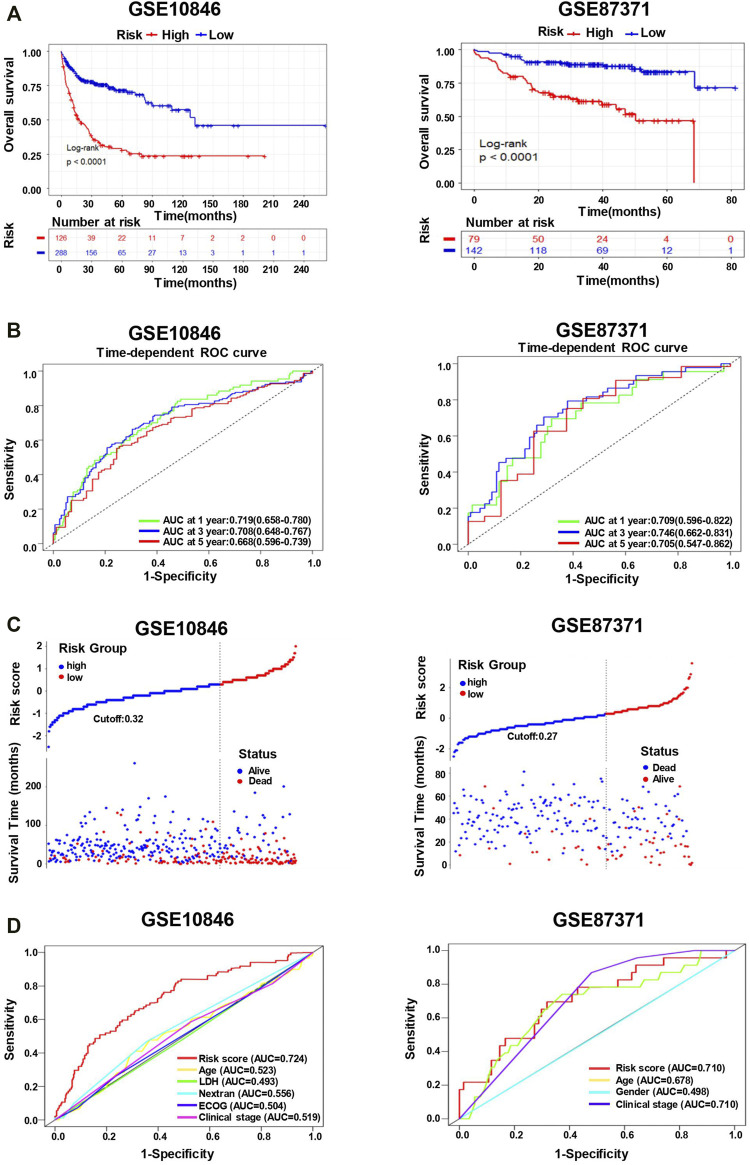
The 11-gene risk score model for the validation datasets (GSE10846 and GSE87371). **(A)** Kaplan–Meier plots of overall survival in high-risk and low-risk subgroups in the validation datasets derived *via* Log-rank testing. **(B)** The time-dependent ROC curve and AUC in the validation datasets. **(C)** The survival status and time in high-risk and low-risk groups for the validation datasets. **(D)** The ROC curves to predict the sensitivity and specificity of clinicopathological factors and 11-gene signature-derived risk scores in DLBCL patients for the validation datasets.

To further validate the effect of the prognostic predictive model, we analyzed two independent external datasets, GSE10846 and GSE87371, with a similar working procedure as mentioned above. The detail of the clinical characteristics is also described in Supplementary Table S1. The risk scores of each cohort were calculated, which divided the patients into low- and high-risk groups. As the consistent result of the two datasets shown in Figure 6A, the overall survival was distinguished from different groups in K–M analysis (long-rank test, *p* < 0.0001). In addition, the time-dependent ROC curve analyses also performed favorable outcomes, in which the AUC of 1-year at 0.719, 3-year at 0.708, 5-year at 0.668 in GSE10846, and the AUC of 1-year at 0.709, 3-year at 0.746, 5-year at 0.705 in GSE87371 (Figure 6B). When the cutoff points were 0.32 and 0.27 in GSE10846 and GSE87371, respectively, the patients were separated into low- and high-risk groups subsequently. There were more death events in the high-risk group from both datasets (Figure 6C). Moreover, we also conducted the ROC curve analyses to evaluate the predictive performance of the 11-genes risk score model and some other clinical variables. The AUCs of the risk score were 0.724 in GSE10846 and 0.710 in GSE87371, significantly greater than that of any other clinical parameters (Figure 6D). The univariate and multivariate Cox regression analyses were also used for the two datasets, as shown in Table 1, and the outcome is consistent with the training dataset.

### Chemotherapy Response With Super-Enhancer-Associated Genes Signature

**FIGURE 7:**
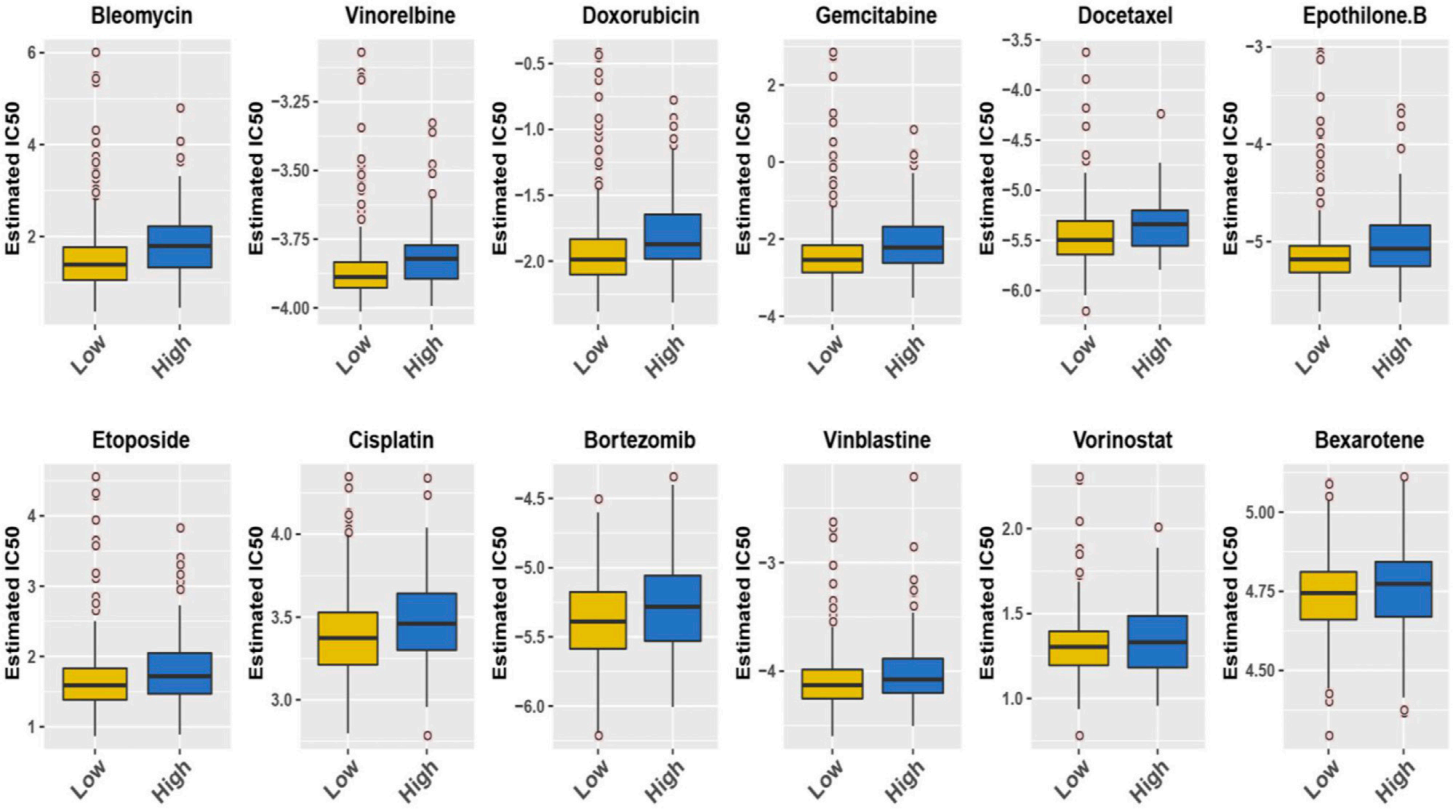
The IC50s of 12 common chemotherapeutic agents with 11-SE-associated-gene signature.

**FIGURE 8:**
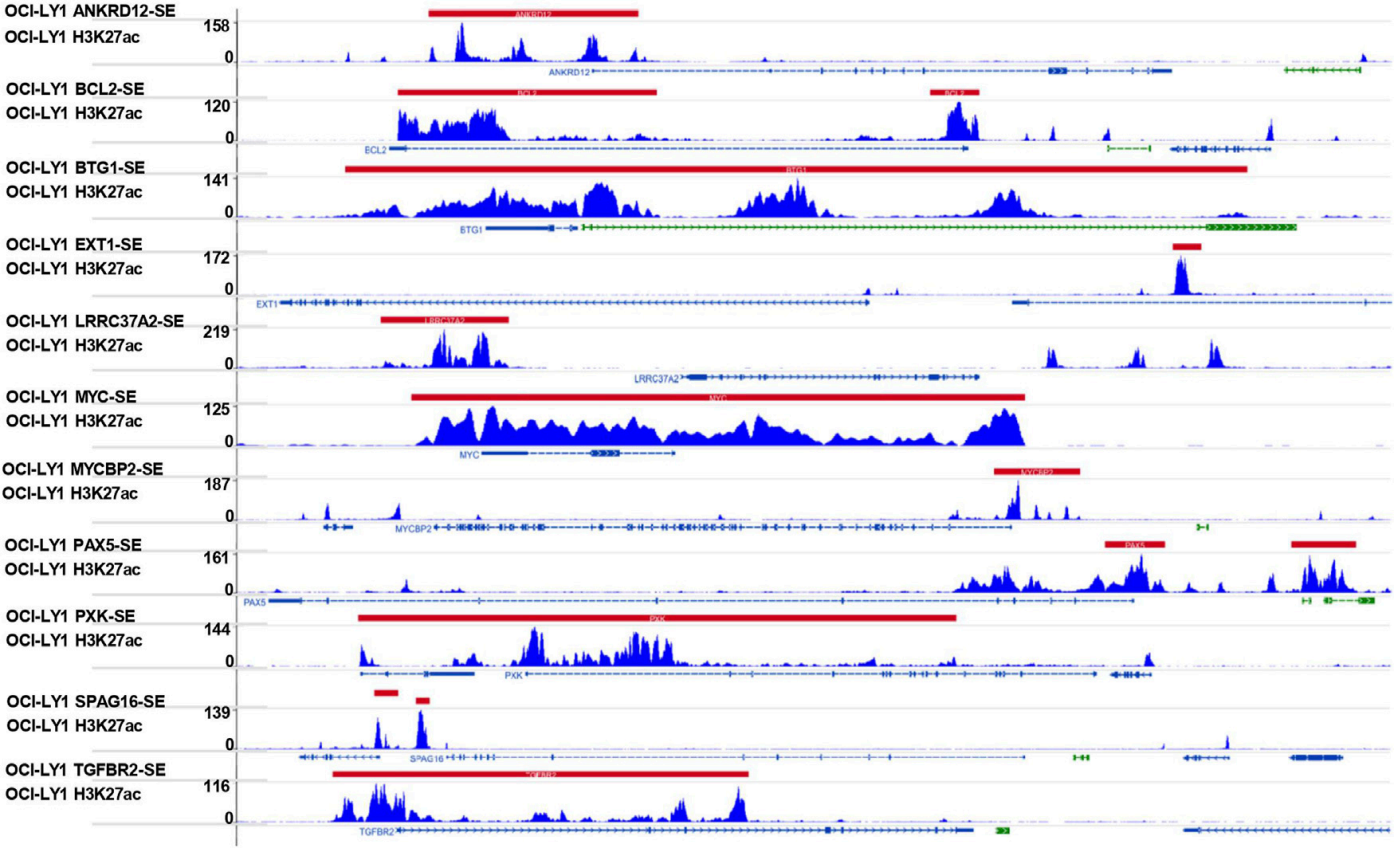
Signal tracks for H3K27ac ChIP–seq profiles of the 11-SE-associated hub genes visualized using IGV. The regions of SE are shown in a red bar upon the signal tracks. ChIP–seq, chromatin immunoprecipitation–sequencing; SE, super-enhancer; IGV, Integrative Genomics Viewer.

In addition, we conducted a prediction analysis to evaluate the chemotherapy response in the low- and high-risk groups. Widely, all high-risk groups possessed higher estimated IC_50_ for the different chemotherapeutics, which exactly proved the hypothesis that the high-risk group was not sensitive to the chemotherapy as the low-risk group (Figure 7). We took 12 chemotherapy drugs into account: bleomycin, vinorelbine, doxorubicin, gemcitabine, docetaxel, epothilone B, etoposide, cisplatin, bortezomib, vinblastine, vorinostat, and bexarotene. In order to better improve the tricky problem, we additionally established an interactive network among these hub genes and chemicals to probe into the chemicals correlated to these genes by “NetworkAnalyst 3.0”. In total, six genes of these 11 hub genes interacted with JQ-1, a well-recognized SE inhibitor, which verified the regulating effect of SEs on these genes to some degree (Supplementary Figure S2B). In the end, we profiled the ChIP signal of H3K27ac-seq for these 11 genes (Figure 8). The predicted regions of SE were plotted as the red bar upon the signal tracks, and each of the predicted SEs located close to these 11 genes, suggesting that the SEs may play an influential role in the expression of the 11 genes. In addition, the SE inhibitor JQ1 may regulate the expression pattern in OCI-LY1 cells.

## Discussion

DLBCL is the most common lymphoma with high heterogeneity and invasiveness. It accounts for approximately one-third of the non-Hodgkin lymphoma, and plenty of patients suffer from insensitive to the typical treatment regimens (Lavacchi et al., 2021). Researchers aspired to identify optimal biomarkers and then establish various risk prediction models for predicting the survival rate, which can be used to improve the prognosis of DLBCL and contribute to personalized therapeutic decisions (Merdan et al., 2021). Enhancer is an important epigenetic regulatory element for DLBCL, which can determine the gene expression. Super-enhancers (SEs) are a large cluster of active enhancers critical for maintaining cell identity and driving the expression of some oncogenes (Kai et al., 2021; Zhou et al., 2021). However, the previous studies had rarely constructed a risk prediction model based on SE-associated hub genes (Li, Duan and Hao, 2021). In this study, we succeeded in building a superior polygenic prognostic model by analyzing the data of the DLBCL patients from the GEO database, taking some clinical indicators into account as well, which was also rare in previous studies.

In the current study, Lasso penalized Cox regression was conducted to identify the candidate SE-associated genes, as the method has recently been prevalent in much research according to its ability to minimize overfitting (Zhu et al., 2019). In addition, we utilized univariate and multivariate Cox regression analyses to narrow the range of the selected genes. Then, we successfully constructed the gene risk score model for survival prediction. Moreover, we integrated the risk score and some other clinical indicators into developing the predictive nomogram and Cox proportion hazards model, which validated the predictive efficacy of the prognostic model. In our study, a total of 417 genes were filtered out by the Lasso penalized Cox regression. Subsequently, two best-fit values (lambda.min and lambda.1se) were chosen, and then the 48-gene group of λ_min_ and 16-gene group of λ_1se_ were initially screened out, respectively. Compared with the result of the AUC and Wilcoxon test, both models performed well. Furthermore, 11 genes were selected when statistically significant both in univariate and multivariate Cox regression analyses. To explore the influence of the 11 candidate genes on the OS and PFS of DLBCL patients, the patients were classified into two groups based on the 11-gene risk score model. The high-risk group had prominent inferior outcomes both in the K-M survival curve and AUC. Combined with some clinical indicators, the univariate and multivariate Cox regression analyses and AUC were conducted to verify the independence of the risk score. Overall, the constructed 11-genes prognostic model demonstrated good predictive performance in the training dataset GSE31312 and the other two external validation sets, GSE10846 and GSE87371. In the training set, BCL2, SPAG16, LRRC37A2, TGFBR2, ANKRD12, and MYC appeared to be the risky factors, apparently upregulated, while PXK, BTG1, EXT1, MYCBP2, and PAX5 were downregulated in high-risk DLBCL patients.

BCL2 is considered an apoptosis suppressor gene. BCL2 is a cell survival protein that inhibits apoptosis by interacting with Bax, Bak, and other pro-apoptotic sensitizer proteins (Nabar et al., 2018) and also contributes to tumorigenesis by its promotion for survival, which already has a long and in-depth research history (Oltersdorf et al., 1998). Currently, many studies have shown a tight correlation between BCL2 expression levels in hematopoietic malignancies and drug resistance during therapy (Stewart et al., 2021). Previous studies have shown that DLBCL patients overexpressing the BCL2 protein may be strongly related to inferior survival and resistance to the standard therapy (de Jong et al., 2019). BCL2 is an important independent prognostic factor for DLBCL, consistent with our finding that the expression of BCL2 was significantly upregulated in the high-risk groups.

SPAG16 is a gene encoding sperm-associated antigen 16 that plays a role in sperm flagella function and motile ciliogenesis (Zhang et al., 2017; Alciaturi et al., 2019), correlated with the gene expression machinery of germ cells (Nagarkatti-Gude et al., 2011). Siliņa et al. (2011) have proposed that SPAG16 can be a novel autoantibody target and serologic biomarker for cancers. Our study suggested that SPAG16 appears to be an independent predictor, but the specific mechanism to mediate tumorigenesis and its vulnerability to being an immunotherapeutic target remain unknown.

LRRC37A2 is a member of the LRRC37 gene family which is involved in the regulation of protein–ligand interactions and mapped to chromosome 17q21.31-q21.32 (Giannuzzi et al., 2013). Several studies suggested that LRRC37A2 is implicated in epilepsy, epileptic encephalopathy, and Parkinson’s disease, while the effect on DLBCL has never been reported (Yao et al., 2021). In this study, high expression of LRRC37A2 corresponds with an inferior survival outcome that merits further exploitation.

TGFBR2 encodes a protein named transforming growth factor-beta (TGF-β) receptor type 2. This receptor can transduce signals into the intracellular environment, triggering various responses such as cell proliferation, differentiation, motility, and apoptosis (Biswas et al., 2008). Previous studies have shown that acquisition of TGFBR2 somatic mutation may increase the risk of various tumorigenesis and different diseases (Li et al., 2020). This is in line with our result that high-risk patients have upregulated expression of TGFBR2 compared with the low-risk group.

ANKRD12 encodes a 224 kDa nuclear protein ankyrin repeat domain 12, also called ANCO-2. It has been reported that ANCO proteins can inhibit the transcriptional activity of nuclear receptors involved in carcinogenesis (Bai et al., 2013). As per our result, ANKRD12 can predict survival outcomes for DLBCL patients independently, but further investigation is needed to validate.

MYC, well-known as a key transcriptional effector that modulates cellular proliferative and metabolism in stem cells (MacDonald et al., 2010), is also involved in the diverse cellular processes such as adhesion, apoptosis, and DNA damage response, playing a role in the oncogenic effect (Finley et al., 2015). There has been an explosion of molecular, cellular, and animal experiments to illuminate the effect of MYC in the initial development of neoplasms. As for DLBCL patients, MYC rearrangement (MYC-R) may forebode poor prognostic. Rosenwald A et al. have evaluated a large cohort suggesting the adverse prognostic impact of MYC-R and the significant therapeutic potential in DLBCL (Rosenwald et al., 2019). This statement is corroborated again by our study.

As for the protective prognostic factors in our study, PXK encoding protein is involved in ligand-induced internalization, synaptic transmits, and degradation of epidermal growth factor receptors associated with some autoimmunity diseases (Takeuchi et al., 2010). B-cell translocation gene 1 (BTG1) belongs to an anti-proliferative gene family, which regulates autophagy and the cell cycle and is also implicated in DNA repair and mRNA stability (Xue et al., 2021). BTG1 is a well-characterized tumor suppressor for both solid tumors and hematopoiesis and recently has been reported to have a novel role in genotoxic and integrated stress responses. It is evident that the expression level of BTG1 is regarded as a prognostic biomarker for diverse cancers (Yuniati et al., 2019). EXT1 gene produces the protein exostosin-1, which is found in the Golgi apparatus. This protein can modify newly produced enzymes and some proteins, which are critical for metastasis of cancer cells (Francannet et al., 2001). MYCBP2 encodes a ubiquitin (Ub) E3 ligase, which is essential for neurodevelopment (Mabbitt et al., 2020). The antitumor effect of this gene has been identified in various cancers. PAX5 is a member of the paired-box family of transcriptional factors, exclusively expressed in the B-cell lineage (Berek et al., 2008). This gene correlates with a heterogeneous subset of B cell non-Hodgkin lymphoma (B-NHL). The expression level and bio function of Pax5 play a role in normal B lymphopoiesis and prevent tumorigenesis (Medvedovic et al., 2011). The antitumor effect of the above genes is consistent with this study; every gene act as an independent protective prognostic factor, upregulated in the low-risk group. However, the concrete bio function and corresponding molecular machinery of each gene remain a ripe area for further investigation.

Since BCL2, SPAG16, LRRC37A2, TGFBR2, ANKRD12, MYC, PXK, BTG1, EXT1, MYCBP2, and PAX5 are SE-associated genes, the roles of the genes SPAG16, LRRC37A2, ANKRD12, PXK, and BTG1 have not been illuminated in DLBCL, which merits further in-depth analysis in the wet laboratory. In addition, to further assess the efficacy of the 11-gene risk model, large-scale prospective cohorts are still needed.

## Conclusion

In summary, we succeeded in constructing a novel and reliable SE-associated-gene signature that can effectively classify DLBCL patients into high-risk and low-risk groups and perform well in predicting the overall survival. The prediction model can be used as a biomarker of prognosis for DLBCL, which may be a potential therapeutic target and can assist clinicians in the treatment of DLBCL.

## Data Availability

The datasets provided in this study can be obtained from online repositories. The names of the repository/repositories and accession number(s) can be found in the article/Supplementary Material.

## Glossary

ABC: activated B cell
AIC: Akaike information criterion
AUC: area under the ROC
B-NHL: B cell non-Hodgkin lymphoma
ChIP-seq: chromatin immunoprecipitation sequencing
CI: confidence interval
C-index: concordance index
CTD: Comparative Toxicogenomics Database
DLBCL: diffuse large B-cell lymphoma
ECOG: Eastern Cooperative Oncology Group
GCB: germinal center B cell
GEO: Gene Expression Omnibus
GO: Gene Ontology
HR: hazard ratio
IC_50_: half-maximal inhibitory concentration
IGV: Integrative Genomics Viewer
IHC: immunohistochemical
IPI: International Prognostic Index
KEGG: Kyoto Encyclopedia of Genes and Genomes
K–M: Kaplan–Meier
Lasso: least absolute shrinkage and selection operator
LDH: lactate dehydrogenase
MYC-R: MYC rearrangement
NHL: non-Hodgkin’s lymphoma
OS: overall survival
PFS: progression-free survival
R-CHOP: rituximab, cyclophosphamide, doxorubicin, vincristine, and prednisone
ROC: receiver operating characteristic
SE: super-enhancer
TE: typical-enhancer
Ub: ubiquitin
UC: unclassified
