# Supplementary material for: A Novel Defined Super-Enhancer Associated Gene Signature to Predict Prognosis in Patients With Diffuse Large B-Cell Lymphoma
Source: Front Genet. 2022 Jun 14;13:827840. doi: 10.3389/fgene.2022.827840 (PMC9237400; doi:10.3389/fgene.2022.827840)
Supplement: Supplementary file 2 [file DataSheet1.docx]

**Supplementary Figures**


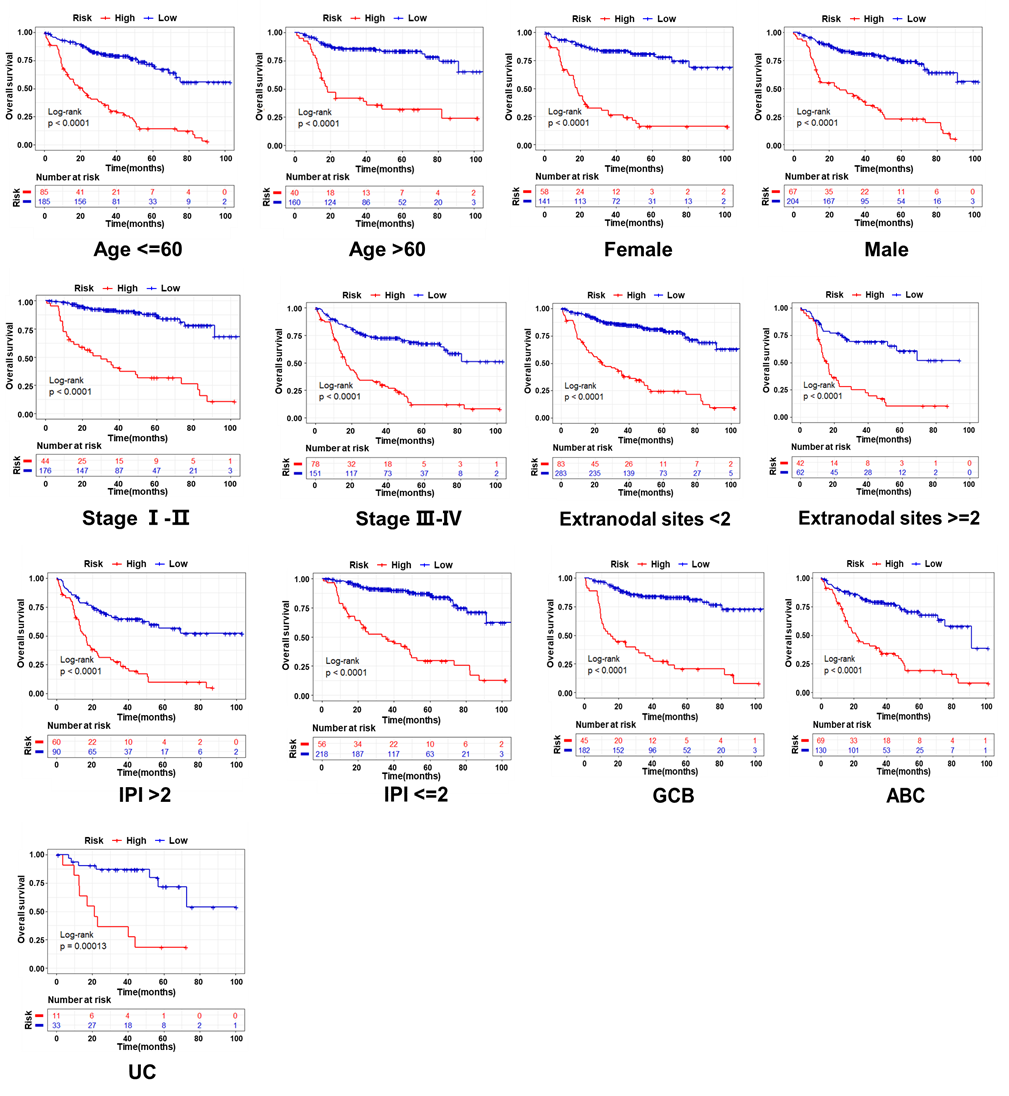


**Supplementary Figure S1 |** Kaplan-Meier analysis of overall survival for DLBCL patients stratified by age, gender, stage, extra nodal invasion, IPI score and histological type. Red is the high-risk group and blue is the low-risk group.


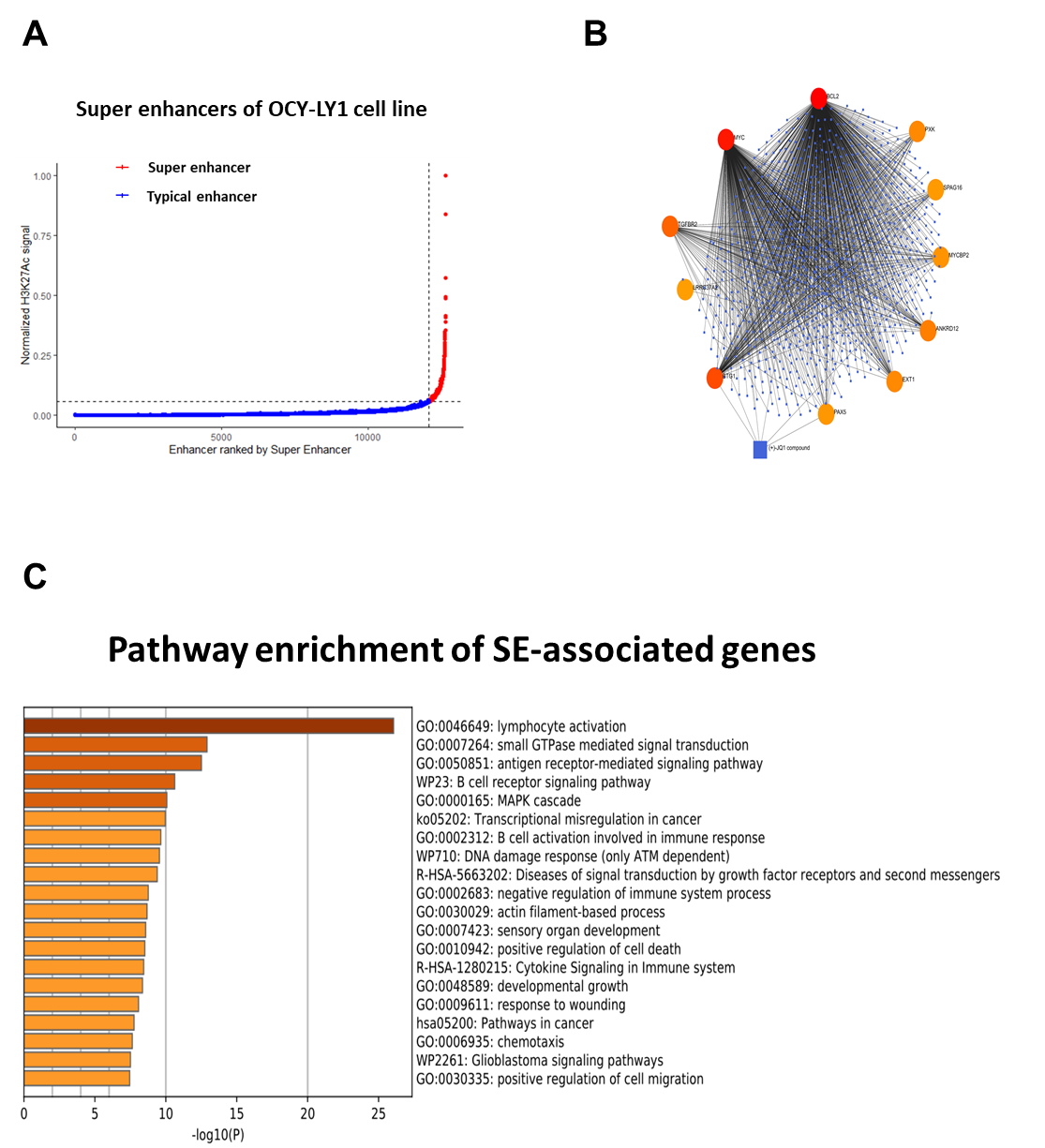


**Supplementary Figure S2 |** Pathway enrichment analysis of SE-associated genes. **(A)** Identification of super-enhancers in OCI-LY1 cell line by ROSE algorithm. Enhancers were ranked and plotted by H3K27ac ChIP-seq signal. Red dots represent super enhancers. Blue dots represent typical enhancers. **(B)** Gene–drug interaction network analysis of the eleven genes and drugs. **(C)** Pathway enrichment analysis of SE-associated genes in OCI-LY1 cell line was performed on the website (https://metascape.org/gp/index.html#/main/step1).
